# Supplementary material for: Evidence supported by Mendelian randomization: impact on inflammatory factors in knee osteoarthritis
Source: Front Med (Lausanne). 2024 May 28;11:1382836. doi: 10.3389/fmed.2024.1382836 (PMC11165061; doi:10.3389/fmed.2024.1382836)
Supplement: Supplementary file 1 [file Data_Sheet_1.ZIP › data/GCST90274787_5e6/787.leaveoneout.pdf]

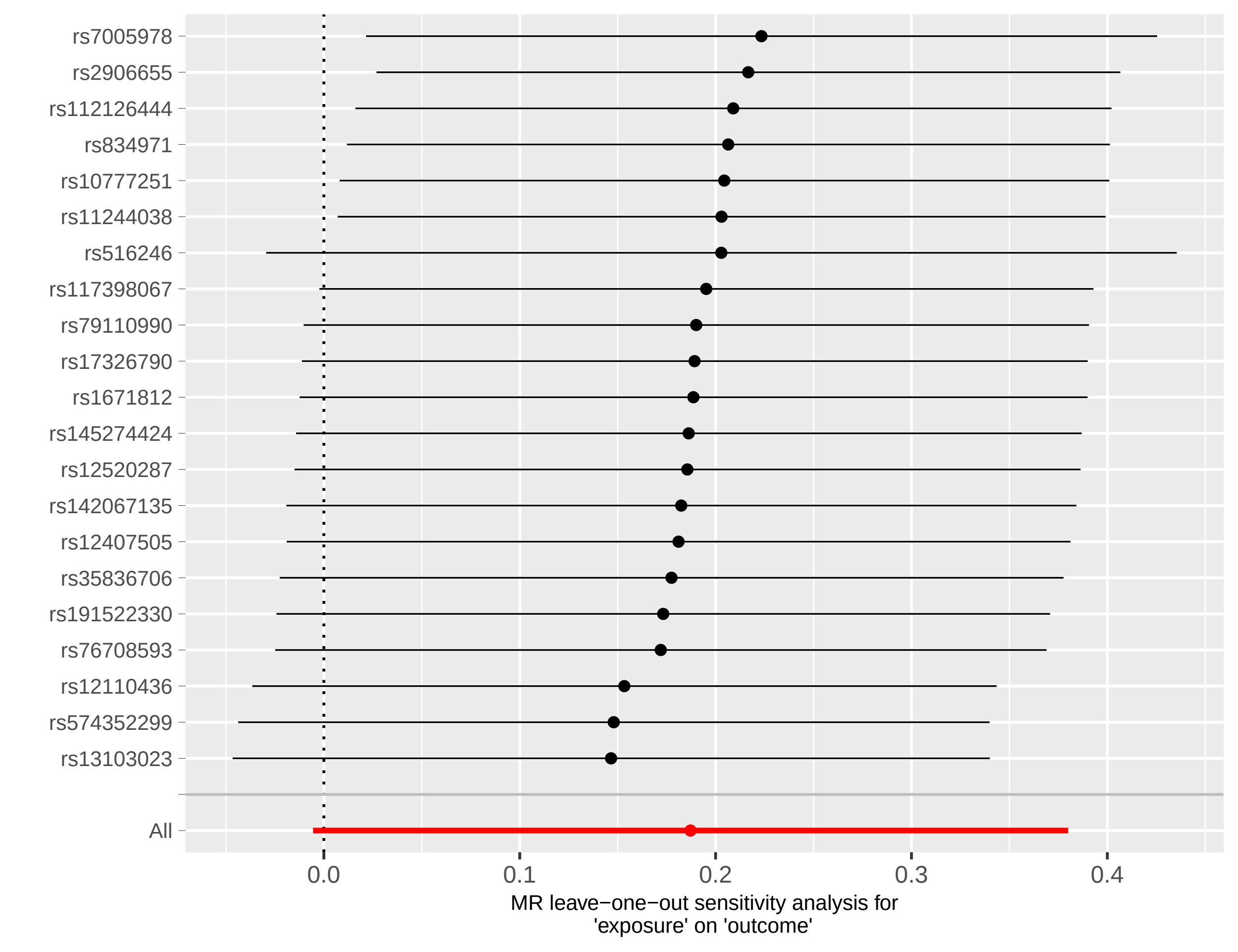
rs7005978
rs2906655
rs112126444
rs834971
rs10777251
rs11244038
rs516246
rs117398067
rs79110990
rs17326790
rs1671812
rs145274424
rs12520287
rs142067135
rs12407505
rs35836706
rs191522330
rs76708593
rs12110436
rs574352299
rs13103023
All
0.0
0.1
0.2
0.3
0.4
MR leave−one−out sensitivity analysis for
'exposure' on 'outcome'
